# 

**Published:** 1858-09

**Authors:** 


### CONTENTS OF VOL. XII.

Address on Artificial Dentures. By Dr. J. Allen........ 35
American Dental Convention—Fourth Annual Session........ 61
A Synopsis—Dental Ethics. By J. Taft........ 173
A High Standard of Attainment—the Demand of the Age. By B. P. Aydelott, D.D........ 253
American Dental Convention—Editorial........ 123
An Address, By W. W. Allport........ 262
A Clamp. By J. Taft........ 377
A Brief Review of Dental Associations. By A Senior........ 438
A New Method of Mounting Teeth—Editorial........ 455
Atmospheric Pressure Plates........ 448
A Case of Fracture of the Lower Jaw at its neck—Miscellaneous........ 104
Blindness caused by Engorgement of the Maxillary Sinus, read before the Medical Society of Beaver Co. By J. Murray, Surgeon Dentist, Beaver, Pa........ 32
Bonsall's Address........ 165
Basis of a National Dental Association. By J. G Hamill........ 369
Continuous Gum Work. By Dr. W. B. Roberts........ 130
Criminal Assault by a Dentist........ 212
Chloroform........ 230
Cleanliness........ 347
Case of an Adult never furnished with Teeth........ 352
Cincinnati Dental Association—Editorial........ 362
Correspondence—Alex. Robertson........ 416
Dental Quacks. By H. R. Smith........18, 179, 343
Dental Colleges and Honorary Degrees........ 349
Dental Advertisements. By J. Richardson........ 378
Extracting Teeth—Electro-Magnetism—Editorial........ 235
Editorial........119, 239, 353, 445
Ether Question—Editorial........ 240
Filling Teeth. By J. Taft........5, 125
Fourth Annual Meeting of the American Dental Convention—Discussions........ 70
Fifteenth Annual Meeting of the Mississippi Valley Association of Dental Surgeons........ 306
Fifteenth Annual Meeting of the Mississippi Valley Association—Discussions........ 320
Galvanism in Extracting Teeth—Editorial........ 119
Gold. By Prof. Austin........ 430
How are Good Teeth Secured ? By E. Collins........ 12
How to fix your Forceps before Extracting Teeth........ 189
How does Chloroform cause Death ? By T. S. Maddin, M. D........ 192
How Teeth are Destroyed........ 203
History of the Ohio College of Dental Surgery........ 336
Irregularities in the Number of Teeth. By H. H. Shadoan........ 16
Introductory Lecture to the Dental Class of 1858-9. By Prof. C. B. Chapman........ 170
Is Electricity Beneficial in Extracting Teeth ? By O. W. True........ 374
Kellum's Soldering Flask........ 190
Lateral Pressure of the Teeth considered in relation to obscure Nervous Affections, read at the Fourth Annual Meeting of the "American Dental Convention." By J. McCalla........ 43
Miscellaneous........104, 232, 452

CONTENTS.

Management of Gold Alloy. By Geo. Watt.......... 245
Minutes and Discussions of the Indiana State Convention.......... 281
Minutes of the Ohio Dental College Association.......... 303
Modus Operandi of Chloroform.......... 437
On the Artificial Production of Bone. By. Dr. Leopold Ollier.......... 435
Obituary.......... 458
Porcelain Teeth. By Daniel L. Pratt.......... 185
Proceedings of Societies—Cincinnati Association of Dental Surgeons.......... 381
Professional Ethics. By J. Taft.......... 422
Painless Surgery—Editorial.......... 358
Remarks on the Best Means of Securing Good Teeth, read before the "American Dental Convention," Cincinnati. By J. Richardson.......... 24
Remarks on Leeches. By Frederick Stearns.......... 201
Report on Dental Progress to the Mississippi Valley Association of Dental Surgeons.......... 310
Signs of Lesions of Nerve Centers. By Prof. C. B. Chapman.......... 170
Splitting of the Alveolar Process of the Lower Jaw.......... 187
Soluble Quartz. By Geo. Watt.......... 363
The Philosophy of Extracting Teeth by Electricity without Pain.......... 350
Treatment of Fractures of the Lower Jaw. By W. W. Allport.......... 230
Treatment of Periostitis and Alveolar Abscess. By E. Collins.......... 366
Treatment in a case of an Over Dose of Chloroform.......... 429
The Employment of Dentists in the Army.......... 450
Third Annual Meeting of the Northern Ohio Dental Convention.......... 388
The American Journal on Dental Chemistry—Editorial.......... 237
The American Journal—Editorial.......... 353
The American Journal and the American Dental Convention—Editorial.......... 239
The Best Means of Securing Good Teeth—Miscellaneous.......... 14

TWELFTH VOLUME

OF THE

### DENTAL REGISTER OF THE WEST.

EDITED BY

J. TAFT and G. WATT.

TERMS: - - - - - - - - $3 PER ANNUM, IN ADVANCE.

### Advertisements Inserted as follows:

One page, per annum,.......................................................$25 00
One-half page, per annum..................................................... 15 00
Quarter page, per annum,..................................................... 10 00

Shorter periods than one year, by special arrangement.

☞Contributions to the pages of the *Register* respectfully solicited.

Exchanges and Correspondents will please address "Dental Register," or "J. Taft, Cincinnati, Ohio."

### PROCEEDINGS OF SOCIETIES.

American Dental Convention—Fourth Annual Session,.............................. 61
Fourth Annual Meeting of the American Dental Convention. Discussions,............ 70

Miscellaneous.............................................................................. 104

Editorial.................................................................................... 119

